# Impaired noradrenaline homeostasis in rats with painful diabetic neuropathy as a target of duloxetine analgesia

**DOI:** 10.1186/1744-8069-9-59

**Published:** 2013-11-27

**Authors:** Jun Kinoshita, Yukari Takahashi, Ayako M Watabe, Kazunori Utsunomiya, Fusao Kato

**Affiliations:** 1 Department of Neuroscience, Jikei University School of Medicine, Minato, Tokyo 105-8461, Japan; 2 Division of Diabetes, Metabolism and Endocrinology, Department of Internal Medicine, Jikei University School of Medicine, Minato, Tokyo 105-8461, Japan; 3 Nagoya University Graduate School of Medicine, Nagoya 466-8550, Japan

**Keywords:** Pain, Streptozotocin, Diabetes mellitus, Noradrenaline, DSP-4, Duloxetine, Spinal cord, Dopamine-beta-hydroxylase, Norepinephrine transporter

## Abstract

**Background:**

Painful diabetic neuropathy (PDN) is a serious complication of diabetes mellitus that affects a large number of patients in many countries. The molecular mechanisms underlying the exaggerated nociception in PDN have not been established. Recently, duloxetine (DLX), a serotonin and noradrenaline re-uptake inhibitor, has been recommended as one of the first-line treatments of PDN in the United States Food and Drug Administration, the European Medicines Agency and the Japanese Guideline for the Pharmacologic Management of Neuropathic pain. Because selective serotonin re-uptake inhibitors show limited analgesic effects in PDN, we examined whether the potent analgesic effect of DLX contributes toward improving the pathologically aberrant noradrenaline homeostasis in diabetic models.

**Results:**

In streptozotocin (STZ) (50 mg/kg, i.v.)-induced diabetic rats that exhibited robust mechanical allodynia and thermal hyperalgesia, DLX (10 mg/kg, i.p.) significantly and markedly increased the nociceptive threshold. The analgesic effect of DLX was nullified by the prior administration of N-(2-chloroethyl)-N-ethyl-2-bromobenzylamine (DSP-4) (50 mg/kg, i.p.), which drastically eliminated dopamine-beta-hydroxylase- and norepinephrine transporter-immunopositive fibers in the lumbar spinal dorsal horn and significantly reduced the noradrenaline content in the lumbar spinal cord. The treatment with DSP-4 alone markedly lowered the nociceptive threshold in vehicle-treated non-diabetic rats; however, this pro-nociceptive effect was occluded in STZ-treated diabetic rats. Furthermore, STZ-treated rats exhibited a higher amount of dopamine-beta-hydroxylase- and norepinephrine transporter-immunopositive fibers in the dorsal horn and noradrenaline content in the spinal cord compared to vehicle-treated rats.

**Conclusions:**

Impaired noradrenaline-mediated regulation of the spinal nociceptive network might underlie exaggerated nociception in PDN. DLX might exert its analgesic effect by selective enhancement of noradrenergic signals, thus counteracting this situation.

## Background

Diabetic neuropathy is one of the most frequent complications of diabetes mellitus (DM) [1]. Of the major symptoms associated with diabetic neuropathy, exaggerated pain crucially impairs the physical and mental components of quality of life in a large number of patients with types 1 and 2 DM [2,3]. This complication of DM, often called painful diabetic neuropathy (PDN), is characterized by allodynia, which is an aberrant painful sensation to normally innocuous stimuli, and hyperalgesia, which is an increased sensitivity to painful stimuli [4,5]. Such exaggerated nociception is also reproduced in animal models of DM, such as the type 1 DM model induced with streptozotocin (STZ), an agent that selectively destroys pancreatic β cells after being taken up through glucose transporter 2 [6]. The animals treated with STZ show, in addition to robust hyperglycemia and hypoinsulinemia, severe chronic pain characterized with decreased mechanical and thermal pain threshold [6]. Despite the presence of these animal models of PDN, the molecular mechanisms underlying the exaggerated nociception in diabetic patients and animal models have not been established [7]. Identifying these mechanisms would facilitate the development of novel and more effective medical interventions for PDN.

Recently, duloxetine (DLX), an antidepressant with a serotonin (5-HT) and noradrenaline (NA) re-uptake inhibitor (SNRI), has been shown to be highly effective in relieving pain in diabetic patients [8-10] and in STZ-treated animal models [11-13]. This pain relief effect is not unique to DLX, and it is possessed by other SNRIs, which also improve the exaggerated pain in diabetic patients [14] and animal models [15,16]. SNRIs, including DLX, are recommended to be one of the first-line treatments for patients with PDN [5,17]. Though there is a high incidence of PDN-depression complication, it is unlikely that this pain-relieving effect of DLX is a simple consequence of the amelioration of depression because DLX relieves neuropathic pain without significantly improving depression in patients with such complication [18,19]. These lines of evidence support the notion that not only the peripheral nerve damage but also a failure in NA/5-HT regulation would underlie the pathogenesis of PDN and this would be the target of DLX. Additionally, because of the absence of a significant effect of selective 5-HT re-uptake inhibitors (SSRIs), which is another group of widely used antidepressants in human patients [20], it is suggested that a modulation of NA homeostasis by DLX underlies its pain relief in DM patients.

Indeed, aberrant NA homeostasis is reported in another form of chronic pain model with peripheral nerve injury [21]. It is possible that hyperglycemia-induced neuropathy [22] and spinal cord inflammation [6], as observed also in the nerve-injury models [23], gave rise to aberrant NA homeostasis also in the PDN models. Interestingly, a recent study suggested that insulin signal is involved in the regulation of NA homeostasis [24]. This finding is of high importance because low-dose insulin itself has potent analgesic effect [25] as well as ameliorative effects on the neuropathy [22]. A possible hypothesis would be that, in the PDN animals, aberrant NA homeostasis resulting from both of hyperglycemia-induced neuropathy and hypoinsulinemia-induced modulation of NA homeostasis would exacerbate the hyperalgesic behaviors. If this is the case, it is expected that rectification of NA homeostasis would potently relieve pain in PDN animals in a manner dependent on recuperation of NA-mediated regulation of spinal nociception.

In the present study, we demonstrate that an improvement in pathologically aberrant NA homeostasis underlies the potent analgesic effect of DLX in diabetic models. To address this issue, we evaluated the effects of DLX on nocifensive behaviors and the expression of molecules for NA homeostasis in the spinal cord in STZ models using histochemical and biochemical approaches; we also examined the effects of the pharmacological deletion of noradrenergic fibers. The results strongly suggest that the mechanisms that regulate the spinal NA levels, presumably arising from the descending pain regulatory system [26,27], become dysfunctional in the PDN models and that DLX exerts its analgesic effect by improving this dysfunction.

## Results

### Blood glucose levels and body weights of the experimental groups

**Figure 1:**
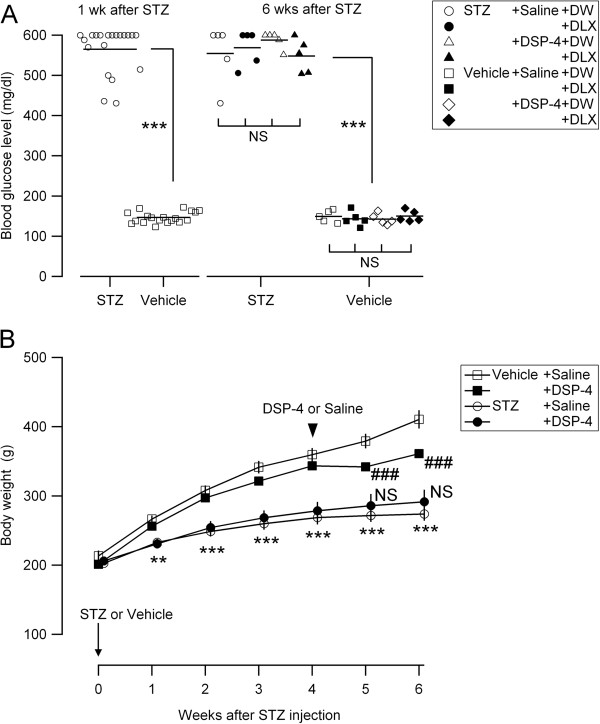
**Metabolic profiles of the experimental groups. (A)** Blood glucose levels for the rats treated with STZ and vehicle. Each marker represents the value measured in each rat belonging to different groups (shown in the inset). The horizontal bars indicate the mean of blood glucose levels for each group. Left, values measured at 1 week (wk) after STZ injection; ***P < 0.001 (Mann–Whitney *U*-test) between STZ-treated (open circles) and vehicle-treated (open squares) groups. Each experimental group was composed of 20 rats. Right, values measured at 6 weeks (wks) after STZ injection; ***P < 0.001 (Mann–Whitney *U*-test) between STZ-treated (circles and triangles) and vehicle-treated (squares and diamonds) groups; NS, not significantly different (Kruskal-Wallis one-way ANOVA) between saline + DW, saline + DLX, DSP-4 + DW and DSP-4 + DLX groups in the rats treated with STZ and vehicle. Each experimental group was composed of 5 rats. **(B)** Time course of body weights of the rats treated with STZ and vehicle. At week 0, the rats received i.v. injection of STZ (n = 20 rats) or vehicle (n = 20) (arrow on the left-bottom). At 4 weeks after STZ injection, the rats received i.p. injection of DSP-4 (n = 10 rats) or saline (n = 10) (black arrowhead). The mean and SEM (vertical bars) values of 10 rats for each experimental group are shown. **P < 0.01; ***P < 0.001 (ANOVA repeated-measures) between STZ-treated (circles) and vehicle-treated (squares) groups; ###P < 0.001 (ANOVA repeated-measures) between vehicle + saline (open squares) and vehicle + DSP-4 (filled squares) groups; NS, not significantly different (ANOVA repeated-measures) between STZ + saline (open circles) and STZ + DSP-4 (filled circles) groups.

Rats treated with STZ (50 mg/kg, i.v.) consistently showed significantly higher blood glucose levels (range, 431–600 mg/dl; n = 20) compared to the rats treated with vehicle (range, 123–172 mg/dl; n = 20) at 1 week after STZ injection (Figure 1A, left). Such hyperglycemia was observed in all of the rats at 6 weeks after STZ injection (Figure 1A, right). In this series, 50% of the rats received N-(2-chloroethyl)-N-ethyl-2-bromobenzylamine (DSP-4) (50 mg/kg, i.p.), a medication that selectively degenerates noradrenergic fibers [28], at 4 weeks after STZ injection. This medication did not significantly affect the blood glucose levels in both STZ- and vehicle-treated groups (Figure 1A, right). The DLX injection (10 mg/kg, i.p.) at 2 hours before blood sampling at 6 weeks after STZ injection did not significantly affect the blood glucose levels (Figure 1A, right). The body weights of STZ-treated rats were significantly lower than those of vehicle-treated rats during 6 weeks of observation (Figure 1B, circles). DSP-4 treatment at the 4th week after STZ injection significantly decreased the body weights at the 5th and 6th weeks in vehicle-treated rats (Figure 1B, filled squares) [29] but not in STZ-treated rats (Figure 1B, filled circles). These observations indicate that all of the rats that received STZ became diabetic, in accordance with previous reports [30,31], and this effect did not occur with the administration of DSP-4 and DLX.

### STZ induced thermal hyperalgesia and mechanical allodynia, which was occluded by pre-treatment DSP-4

**Figure 2:**
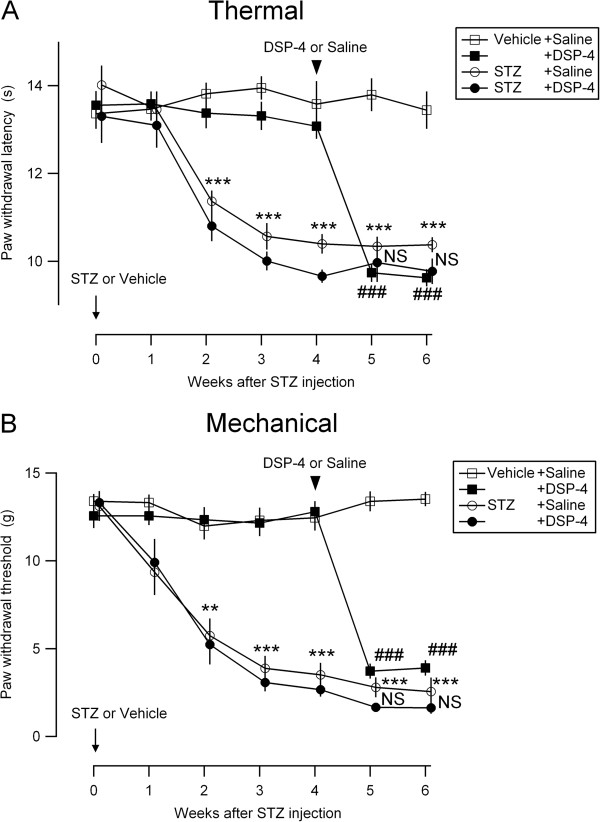
**Effects of STZ and DSP-4 on thermal and mechanical nociception.** Time course of thermal hyperalgesia **(A)** and mechanical allodynia **(B)** in the rats treated with STZ and vehicle. At week 0, rats received an i.v. injection of STZ (n = 20 rats) or vehicle (n = 20) (arrow on the left-bottom). At 4 weeks after STZ injection, the rats received an i.p. injection of DSP-4 (n = 10 rats) or saline (n = 10) (black arrowhead). The mean and SEM (vertical bars) values of 10 rats for each experimental group are shown. **P < 0.01; ***P < 0.001 (ANOVA repeated-measures) between STZ-treated (circles) and vehicle-treated (squares) groups; ###P < 0.001 (ANOVA repeated-measures) between vehicle + saline (open squares) and vehicle + DSP-4 (filled squares) groups; NS, not significantly different (ANOVA repeated-measures) between STZ + saline (open circles) and STZ + DSP-4 (filled circles) groups.

In agreement with previous reports [6,31], the diabetic rats showed increased nocifensive behaviors. The paw withdrawal latency to thermal stimulation (Figure 2A, circles) and the paw withdrawal threshold to mechanical stimulation (Figure 2B, circles) were significantly decreased after the second week of STZ injection and lasted at least for 6 weeks after injection. DSP-4 treatment at the 4th week decreased the latency to thermal stimulation (Figure 2A, filled squares) and threshold to mechanical stimulation (Figure 2B, filled squares) in vehicle-treated animals, similar to previous studies [32,33], suggesting that an impaired integrity of the noradrenergic system leads to exaggerated nocifensive behaviors. Surprisingly, this pro-nociceptive effect of DSP-4 was not observed in STZ-treated animals (Figure 2A and B, filled circles), which indicated that the pro-nociceptive effect of DSP-4 was occluded in STZ-treated diabetic rats.

### Effects of DLX on STZ-induced hyperalgesia/allodynia in DSP-4 pretreated animals

**Figure 3:**
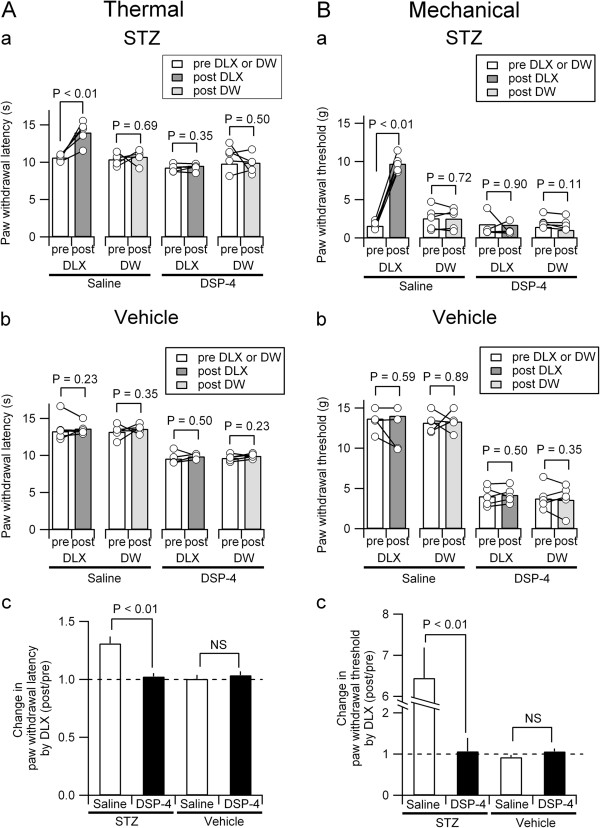
**Effects of DSP-4 on analgesic effects of DLX in STZ-treated rats.** Effects of DLX treatment on thermal hyperalgesia **(A)** and mechanical allodynia **(B)** in the rats treated with STZ **(a)** and vehicle **(b)** at 6 weeks after STZ injection. The control rats received equal volumes of distilled water (DW) instead of DLX. Thermal hyperalgesia was assessed at 60 min before (pre-DLX or DW, white columns) and 60 min after injection of DLX (post-DLX, dark gray columns) or DW (post-DW, light gray columns). Mechanical allodynia was assessed at 30 min before (pre-DLX or DW) and 90 min after injection of DLX (post-DLX) or DW (post-DW). Circles represent the values at pre-DLX and post-DLX (or DW) obtained from each rat. Bars indicate the mean values at pre-DLX and post-DLX (or DW). Each experimental group was composed of 5 rats. The differences in the values of pre-DLX and post-DLX were compared by Wilcoxon signed-rank test. **(c)** Change in paw withdrawal latency **(A)** and threshold **(B)** by DLX in the rats treated with STZ and vehicle. Change in paw withdrawal latency and threshold by DLX (*y*-axis for Ac and Bc) was calculated as the value of post-DLX divided by the value of pre-DLX. The mean ± SEM values of 5 rats for each experimental group are shown. The differences in the values were compared using the Mann–Whitney *U*-test.

DLX, one of the first-choice medications for PDN [5,17], is an inhibitor of 5-HT and NA transporters. The decreased latency to thermal stimulation (Figure 3Aa; compare “Saline” and “DSP-4”) and the lowered threshold for mechanical stimulation (Figure 3Ba) in STZ-treated animals were significantly increased by a single injection of DLX (10 mg/kg, ip), supporting its pain relieving effect in the STZ models of PDN [11-13]. DLX exerted no significant changes in vehicle-treated rats (Figure 3Ab and Bb, “Saline”). In contrast, in the STZ-treated rats that received DSP-4 injection, DLX exerted no significant effect on both the thermal and the mechanical thresholds (Figure 3Aa and Ba, “DSP-4”). This absence of a DLX effect in DSP-4-treated rats was also similarly observed in vehicle-treated rats (Figure 3Ab and Bb, “DSP-4”). In this series of study, we have measured the nocifensive behaviors before and after DLX administration. It is therefore possible to evaluate how DLX improved nociception in individual rats by normalizing the latency (thermal) and threshold (mechanical) after DLX injection by the values measured before DLX injection (Figure 3Ac and Bc). This allowed direct comparison of the efficacy of DLX between in the absence and presence of DSP-4-treatment. The normalized effects of DLX were almost 1.0 (i.e., almost no effect) in STZ-treated rats that received DSP-4 and in vehicle-treated rats with or without DSP-4 (Figure 3Ac and Bc). In contrast, the effects of DLX on both the thermal and the mechanical nocifensive behaviors were significantly greater in STZ-treated rats without DSP-4 injection than in the other groups.

Although to a smaller degree compared to NA fibers, DSP-4 can be uptaken by 5-HT transporters and degenerate serotoninergic fiber terminals [34]. This transport through 5-HT transporters could be prevented by inhibiting 5-HT transporters during the exposure to DSP-4 [28]. To examine whether serotoninergic fiber degeneration is involved in the mechanism of DSP-4 reducing the analgesic effect of DLX, we injected DSP-4 in the presence and the absence of fluoxetine (10 mg/kg, i.p.), a selective 5-HT re-uptake inhibitor, and compared the effect of DLX on nocifensive behaviors. Fluoxetine or saline was administered 30 min before the DSP-4 injection [28]. Regardless of the co-administration of fluoxetine with DSP-4, DLX failed to increase the nociceptive thresholds in STZ-treated rats that received DSP-4 (change in paw withdrawal latency by DLX (post-DLX/pre-DLX): DSP-4 with fluoxetine, 1.01 ± 0.03, n = 5; without fluoxetine, 0.99 ± 0.02, n = 5; P = 0.84; change in paw withdrawal threshold by DLX (post-DLX/pre-DLX): DSP-4 with fluoxetine, 1.12 ± 0.06, n = 5; without fluoxetine, 1.11 ± 0.21, n = 5; P = 0.75) (data not shown in the figure). These results indicate that the major portion of the analgesic effects of DLX in STZ-treated diabetic rats depends on the integrity of spinal noradrenergic systems.

### Effects of STZ and DSP-4 on DBH-immunoreactive fibers in the lumbar spinal dorsal horn

**Figure 4:**
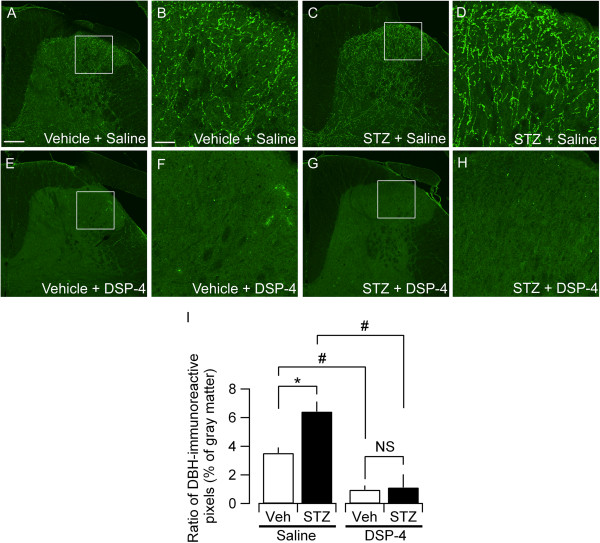
**Effects of STZ and DSP-4 on DBH-immunoreactive fibers in L4-5 dorsal horn.** Representative photomicrographs of DBH-immunoreactive fibers in L4-5 dorsal horn in **(A)** vehicle + saline, **(C)** STZ + saline, **(E)** vehicle + DSP-4 and **(G)** STZ + DSP-4 rats at 6 weeks after STZ injection. Panels **(B)**, **(D)**, **(F)** and **(H)** are higher magnification of boxed area in **(A)**, **(C)**, **(E)** and **(G)**, respectively. The scale bar in **(A)** is 50 μm and applies to **(C)**, **(E)** and **(G)** and the scale bar in **(B)** is 12.5 μm and applies to **(D)**, **(F)** and **(H). (I)** Quantification of DBH-immunoreactive fibers in L4-5 dorsal horn at 6 weeks after STZ injection. Ratio of DBH-immunoreactive pixels to the total pixel number in the dorsal horn gray matter (see Methods). Four to eleven dorsal horn samples were used for evaluating average ratio values in each rat. The mean ± SEM values of 4 rats for each experimental group are shown. *P < 0.05 (Mann–Whitney *U*-test) between vehicle (Veh) and STZ groups in saline-treated animals; #P < 0.05 (Mann–Whitney *U*-test) between saline-treated and DSP-4-treated groups in both STZ and Veh groups; NS, not significantly different.

We therefore analyzed the status of the spinal noradrenergic system in STZ-treated diabetic rats using histochemical and biochemical approaches. Because the nocifensive responses evaluated above (i.e., thermal latency and mechanical threshold) primarily reflect the nociceptor-activated spinal reflex at the level of the lumbar spinal cord, we first evaluated the expression of dopamine-beta-hydroxylase (DBH) protein, an enzyme involved in the conversion of dopamine to NA, using immunohistochemistry analysis of the lumbar spinal dorsal horn of rats treated with STZ and DSP-4. In the dorsal horn of L4-5 spinal cord of the rats treated with vehicle, DBH-immunoreactive fibers were distributed throughout the dorsal laminae (Figure 4A-B). This feature was also observed in the dorsal horn of rats treated with STZ; however, the density of the DBH-positive fibers was higher in the STZ-treated group (Figure 4C-D). The ratio of the DBH-immunopositive pixels to the total number of gray matter pixels as evaluated based on the confocal images of the coronal lumbar sections was significantly larger in the STZ-treated group (Figure 4C-D, I) than in the vehicle-treated rats (Figure 4A-B, I). DSP-4 nearly abolished DBH-immunopositive fibers both in the STZ- and vehicle-treated groups (Figure 4E-H, I), which suggested that DBH-positive fibers were indeed the target of DSP-4. These data suggest that in the STZ-treated group, the production of NA would be increased in the lumbar spinal dorsal horn.

### Effects of STZ and DSP-4 on NET-immunoreactivity in the lumbar spinal dorsal horn

**Figure 5:**
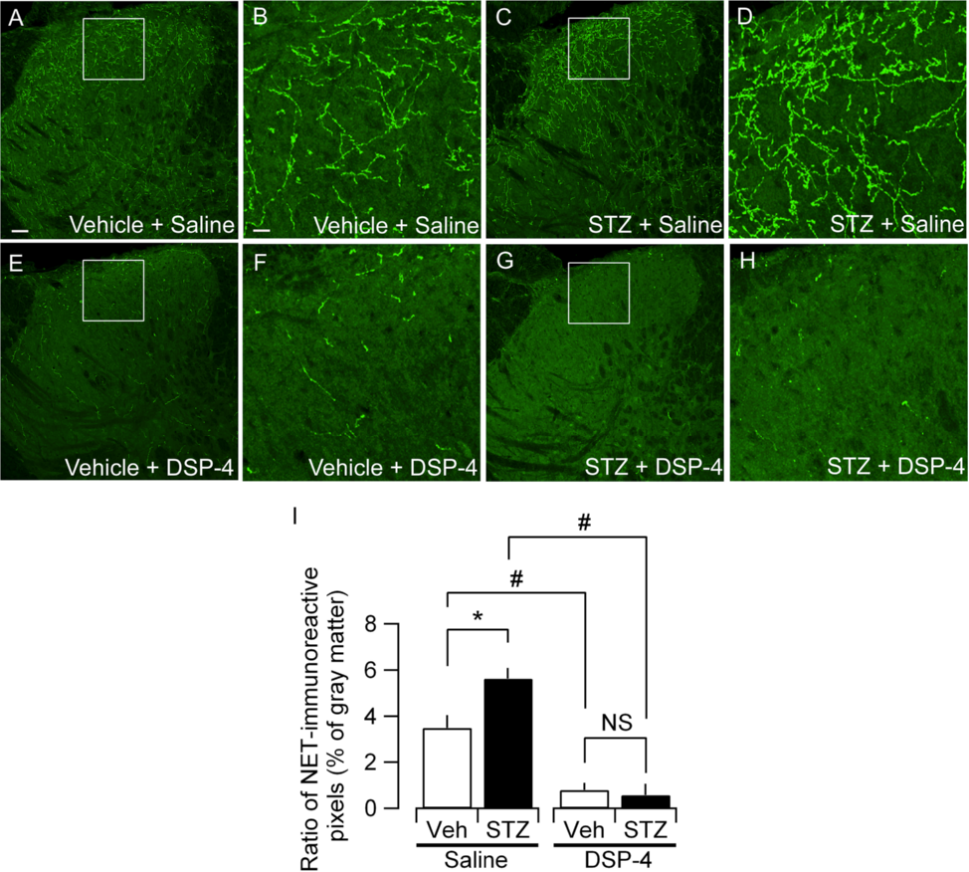
**Effects of STZ and DSP-4 on NET-immunoreactivity in L4-5 dorsal horn.** Representative photomicrographs of NET-immunoreactivity in L4-5 dorsal horn in **(A)** vehicle + saline, **(C)** STZ + saline, **(E)** vehicle + DSP-4 and **(G)** STZ + DSP-4 rats at 6 weeks after STZ injection. Panels **(B)**, **(D)**, **(F)** and **(H)** are higher magnification of boxed area in **(A)**, **(C)**, **(E)** and **(G)**, respectively. The scale bar in **(A)** is 50 μm and applies to **(C)**, **(E)** and **(G)** and the scale bar in **(B)** is 12.5 μm and applies to **(D)**, **(F)** and **(H). (I)** Quantification of NET-immunoreactivity in L4-5 dorsal horn at 6 weeks after STZ injection. Ratio of NET-immunoreactive pixels to the total pixel number in the dorsal horn gray matter (see Methods). Four to twelve dorsal horn samples were used for evaluating average ratio values in each rat. The mean ± SEM values of 4 rats for each experimental group are shown. *P < 0.05 (Mann–Whitney *U*-test) between vehicle (Veh) and STZ groups in saline-treated animals; #P < 0.05 (Mann–Whitney *U*-test) between saline-treated and DSP-4-treated groups in both STZ and Veh groups; NS, not significantly different.

The NA released from noradrenergic terminals was re-uptaken by these terminals and reused. Therefore, the extracellular and intracellular concentrations of NA depend on the activity of this re-uptake. NA is mostly re-uptaken by the Na^+^/Cl^-^-dependent norepinephrine transporter (NET). The genetic ablation of NETs results in an increased extracellular NA level and decreased intracellular storage of NA [35], which indicates that the intra- and extracellular homeostasis of NA depends largely on the activity of NETs. We therefore analyzed the expression of NETs by immunohistochemistry in the lumbar spinal dorsal horn of rats treated with STZ and DSP-4. In rats treated with either STZ or vehicle, NET-immunoreactivity was distributed throughout the dorsal laminae (Figure 5A-D). The quantitative comparison of the pixel density indicated that the fraction of NET-positive pixels was significantly increased in the dorsal horn of STZ-treated rats (Figure 5C-D, I). DSP-4 drastically eliminated the NET-immunoreactivity in the dorsal horn both in the STZ- and vehicle-treated rats (Figure 5E-I). After DSP-4 treatment, there was no significant difference in the ratio of NET-positive pixels between STZ- and vehicle-treated rats (Figure 5I). These results indicated that STZ increased the expression of NETs on the fibers in the lumbar spinal dorsal horn.

### Effects of STZ and DSP-4 on NA content in the lumbar spinal cord

**Figure 6:**
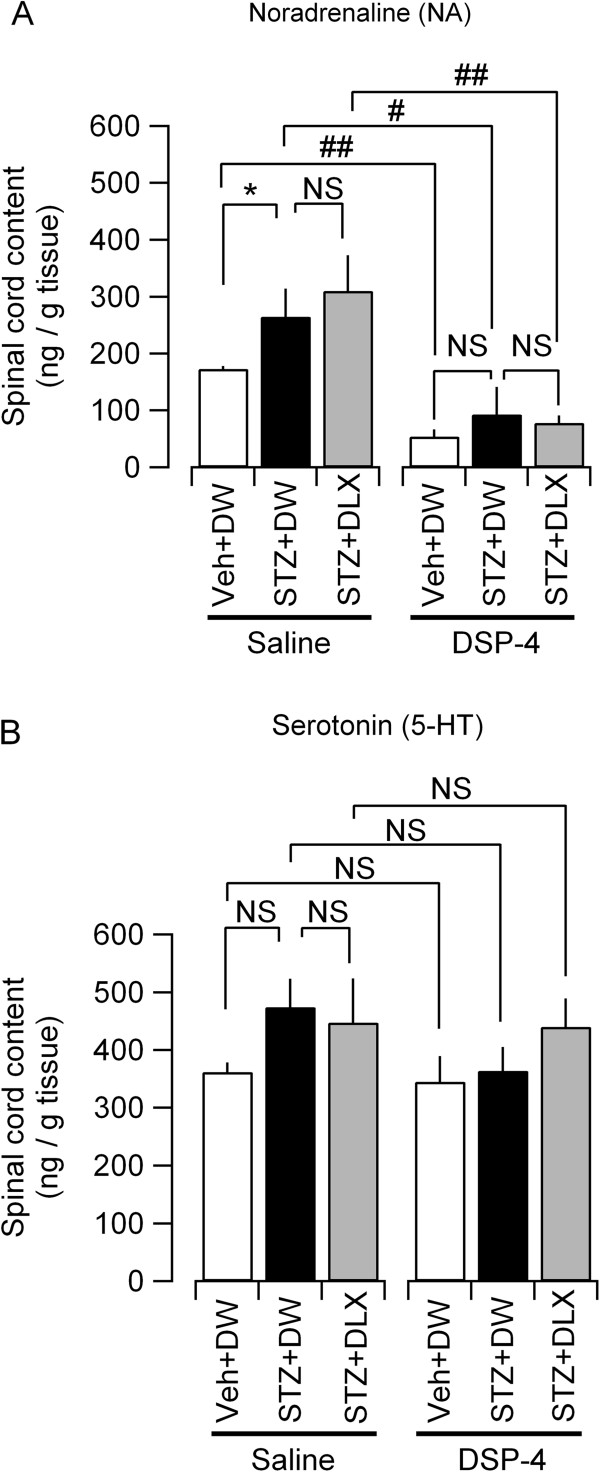
**Effects of STZ and DSP-4 on NA and 5-HT content in the lumbar spinal cord.** NA **(A)** and 5-HT **(B)** content in the lumbar spinal cord at 6 weeks after STZ injection. The mean ± SEM values of 5 rats for each experimental group are shown. The samples were collected from the rats after behavior evaluation (see Methods). *P < 0.05 (Kruskal-Wallis one-way ANOVA) between saline-treated vehicle (Veh) + DW and STZ + DW groups; #P < 0.05; ##P < 0.01 (Mann–Whitney *U*-test) between saline-treated and DSP-4-treated groups; NS, not significantly different.

The increase in DBH- and NET-positive fibers in the lumbar spinal dorsal horn would result in increased NA production and re-uptake into the terminals. To directly confirm this action, we measured the NA content in the lumbar spinal cord tissues from the rats treated with STZ, DSP-4 and DLX using high-performance liquid chromatography (HPLC). We also measured the content of 5-HT using the same homogenized samples that were used for the NA content measurement. STZ significantly increased NA content in the lumbar spinal cord (Figure 6A, “Saline”). DLX did not significantly affect the NA content (Figure 6A, “Saline”). DSP-4 significantly reduced the spinal NA content in rats treated with STZ and vehicle (Figure 6A, “DSP-4”). There was no significant difference in NA content between STZ- and vehicle-treated rats as well as between DLX- and distilled water (DW)-treated rats in the lumbar spinal cord sampled after DSP-4-treatment. Despite slight and insignificant increase in the spinal 5-HT levels in STZ-treated animals, the 5-HT content was not significantly affected by STZ and DSP-4 treatments and was also insensitive to DLX (Figure 6B) unlike the STZ- and DSP-4-induced changes in NA levels (Figure 6A).

## Discussion

Using DSP-4-induced selective ablation of the noradrenergic fibers, we demonstrate that the analgesic effect of DLX in the STZ-induced PDN depends crucially on the presence of intact noradrenergic fibers. Because our analyses indicated drastic changes in the amount of DBH- and NET-expressing fibers in the dorsal horn and the spinal content of NA in the STZ-treated animals, it is highly likely that the potent anti-nociceptive effect of DLX in the STZ-treated animals is mediated by the pharmacological improvement of the pathologically aberrant regulation of spinal NA systems. The mechanisms underlying these effects are discussed below.

### The analgesic effect of DLX depends on an intact NA system

DLX is an SNRI that shows inhibitory potency to NA transporters and 5-HT transporters [36]. The present results support the finding that the analgesic effect of DLX is mediated by its effect on NA transport because the suppression of the DLX effect by DSP-4 pretreatment was clearly observed when DSP-4 treatment was combined with injection of an SSRI (fluoxetine) [28] (see Results). This conclusion that the presence of serotoninergic fibers is not sufficient to produce the anti-nociceptive effect of DLX in PDN is also supported by a recent finding in STZ-treated rats, that the anti-nociceptive effect of another SNRI, venlafaxine, was completely abolished by yohimbine pre-treatment but was only partially inhibited by pretreatment with p-choloroamphetamine, an agent that degenerates serotoninergic fibers [16]. However, partial but significant reduction of analgesic effect of DLX and another SNRI, milnacipran, by 5-HT receptor antagonists has been described in STZ-treated PDN [12] and postoperative pain [37] models of rats. A possible interpretation for these results is that activation of 5-HT receptors might affect extracellular NA concentration, such as through modulating NA release, and that the final common mediator that regulate spinal nociceptive network is the NA system, to which the 5-HT system lies upstream. This is a possibility that might explain why manipulations of either 5-HT or NA system affect the effect of SNRI and eliminating only the NA fibers could completely abolish its analgesic effect, as evidenced in this study. Such a primary role of the NA system in the anti-nociceptive effect of SNRI is also supported by observations in other types of chronic pain models in mice that genetically lack central serotoninergic neurons [38]. In these mice, DLX exerted marked analgesic effects in carrageenan- and formalin-induced pain models to a similar degree as those observed in the wild-type mice, again indicating a secondary involvement of 5-HT system in the analgesic effect of DLX. Altogether, in the chronic model of PDN as used in this study and in other types of chronic pain models, the analgesic effect of DLX requires intact NA systems that are capable of releasing NA from nerve terminals.

### Impaired NA homeostasis would underlie exaggerated nociception in the STZ-diabetic model

This specific modulation of the NA system in the analgesic effect of DLX in STZ-treated rats supports the notion that STZ administration induces long-lasting aberrant modification of the NA systems, which leads to pro-nociception. NA is one of the principal mediators of endogenous analgesic mechanisms in the descending pain modulatory system in the spinal dorsal horn [26,27]. The elimination of NA alone by genetic ablation of DBH or DSP-4 administration potently decreases the nociceptive threshold in mice [39] and rats [32,33], as confirmed in this study. Conversely, intrathecal NA administration increases tail-flick latency in normal mice [40] and rats [41]. Additionally, DSP-4 administration, which drastically increased nociception sensitivity in non-STZ-treated rats, did not further affect the lowered nociceptive threshold in STZ-treated animals in this study (Figure 2). This result is a reminiscence of the absence of otherwise pro-nociceptive effect of 6-hydroxydopamine, an NA synthesis neurotoxin, in STZ-treated mice with lowered nociception threshold [42]. These findings suggest that specific defects in the regulation of NA homeostasis in the spinal cord may underlie the pro-nociception in PDN. In support of this interpretation, the effect of intrathecal NA administration in elevating the nociceptive threshold was markedly more potent in STZ-treated mice than in non-diabetic mice [42]. An increase in the extracellular NA level with DLX would be anticipated because it has been shown, albeit not in a diabetic model, that intravenous injection of milnacipran, which is an SNRI, increases extracellular NA levels in the spinal dorsal horn as measured by microdialysis in anesthetized mice with spinal nerve ligation-induced neuropathy [43]. It is therefore speculated that STZ treatment decreases the spinal NA level, which leads to exaggerated nociception.

Nevertheless, contrary to this speculation, the NA content in the spinal cord was significantly increased in STZ-treated rats in the present study. This result was, however, not unexpected because such an increase in NA in STZ-treated rats is consistent with previous reports [44,45]. In addition to this increase in NA level, the quantities of DBH- and NET-expressing fibers in the dorsal horn were significantly increased in STZ-treated rats. Because these immunopositive fibers in the dorsal horn are drastically abolished after DSP-4 treatment, these molecules are indeed expressed on the segmental branches of the descending noradrenergic fibers.

The NET in the central nervous system is primarily located on the presynaptic membrane of noradrenergic neurons and plays an essential role in the re-uptake of extracellular NA from synaptic clefts to terminals [46,47]. Recently, accumulated lines of evidence point to a clear role of insulin in the regulation of NET expression and membrane localization. The NA uptake in whole brain neuronal culture is inhibited by insulin [48]. The NET mRNA level in the locus coeruleus is reduced by insulin [49] and elevated by STZ treatment [50]. Surface expression of functional NETs in the hippocampal neurons is increased in STZ-treated mice, and conversely, NETs are internalized by acute insulin administration through phosphorylation of Ser/Thr kinase Akt/PKB (protein kinase B) pathways [24].

Our immunohistochemical staining does not allow us to distinguish between surface and internalized NET molecules. However, it is expected that together with the observations of these previous reports, the increased expression of NETs on the fibers in the dorsal horn due to sustained hypoinsulinemia would result in an increased amount of NETs localized on the membrane surface. This activity would lead to an exaggerated NA uptake by the terminals, which leads to decreased extrasynaptic or intracleft NA concentration. Consequently, this decrease in extracellular NA would directly lead to aberrant pro-nociception. The genetic ablation of NETs, which decreases NA content in the spinal cord [51], produces profound hypoalgesia [52]. This insulin-dependent NET expression and the NA dependency of the spinal nociceptive system support the recent view that hypoinsulinemia itself, rather than hyperglycemia, would play a larger role in the establishment of hyperalgesia [53]. Indeed, insulin, at a dose not affecting the hyperglycemia, has been shown to improve neuropathy and relief hyperalgesia [22,25]. Because the NET is the primary target molecule of DLX [54] for its primary effect on NA re-uptake inhibition, the potent anti-nociceptive effect of DLX in STZ-treated rats is, for the most part, attributed to the direct inhibition of exaggerated NA transport in the spinal cord.

Another possibility, which is not incompatible with the interpretation described above, is that the release of NA is lowered in STZ-treated rats. Bitar et al. described a significant reduction in the ratio of 3-methoxy-4-hyroxyphenylglycol (MHPG) to NA in the lumber spinal cord of the rat at 30 days after STZ treatment and suggested a decreased release or turnover of NA in this model [55]. This interpretation is also compatible with the present result of increased NA content in the lumber spinal cord. Decreased NA release would result from decreased firing rate of locus coeruleus neurons and release probability at the spinal noradrenergic axon terminals in STZ-treated rats, possibilities being required to be examined in the future studies.

To date, the molecular mechanisms underlying the increase in the expression of DBH in STZ-treated rats have not been established. The involvement of the CREB pathway in the regulation of tyrosine hydroxylase (TH) [56] and TH expression in STZ-treated diabetic models [45] has been documented. Though it has been shown that increase in brain-derived neurotrophic factor (BDNF) following spinal nerve injury results in sprouting of DBH-expressing fibers in the spinal cord [57], this mechanism is unlikely to primarily underlie the increase in DBH-positive fibers observed in the present study, because the BDNF content in the spinal cord is not significantly affected in a similar PDN model with STZ [58]. In addition to these changes in NA synthesis, the changes in the synaptic expression level of adrenoceptors [55,59] and agonist potency [55,59,60] might also underlie the aberrant NA homeostasis in STZ-treated animals. Whatever the mechanism, increased NA synthesis and storage in the spinal cord in STZ models might result in a larger quantity of NA in the tissue, as evaluated using HPLC (Figure 6). Such augmented NA synthesis and storage would provide support for the effective increase in extracellular NA levels after NET blockade by DLX.

### Mechanisms of anti-nociceptive effect of DLX

We failed to detect a significant increase in lumbar NA level after DLX injection using HPLC analysis (Figure 6) unlike previous studies that demonstrated a significant increase in extracellular NA level induced by DLX in the rat frontal cortex using microdialysis [61,62]. As the expression levels of DBH and the NET were increased in the STZ-treated rats, it is speculated that the changes in extracellular concentration of NA induced by DLX are small compared to the large amount of intracellular stored NA, which obscures the measurement using HPLC. The microdialysis measurement in the dorsal horn in the non-anesthetized animal is a challenging procedure that would provide direct insight into the spinal mechanism of DLX in future studies.

The present results do not necessarily rule out involvement of changes in NA levels in supraspinal structures, such as the limbic system, a pivotal target of nociceptive signals in the brain [63] as well as a site underlying depressive affection. The synaptic transmission of the amygdala neurons, which shows robust synaptic potentiation in chronic neuropathic pain models [64] including STZ-models [65], is modulated by NA [66]. Additionally, the changes in the activities in the amygdala neurons by alpha-2 adrenoceptor agonists affect spinal nocifensive behaviors [67]. These observations might imply that the changes in the amygdala activity by DLX might also underlie these nociceptive effects. Further understanding of the specific molecular facets of supraspinal and spinal NA homeostasis will contribute toward the development of medications with more specific pain-relieving effects in patients with DM.

Clinically, DLX improves pain severity both in type 1 and 2 DM [68]. The PDN model used in this study with STZ treatment mimics the type 1 DM with strong hypoinsulinemia. However, the present finding of the exacerbated spinal nociception through impaired insulin-mediated NA homeostasis might also be of importance in the type 2 DM, in which a larger portion of patients suffer the neuropathic pain [3]. In the animal models for type 2 DM (ob/ob and db/db mice), it has been shown that, despite increased insulin levels, the phosphorylation of Akt is significantly reduced [69]. It is thus expected that such impaired insulin/Akt signal-mediated NA homeostasis would occur and exacerbate nociception also in type 2 DM, which would also be an important target of DLX for its analgesic effect.

We conclude that improvement of extracellular NA homeostasis by inhibiting NETs is the primary mechanism of the anti-nociceptive effect of DLX, which becomes highly potent in painful pathological states that accompany the aberrant increase in NA synthesis and re-uptake in PDN.

## Conclusions

Impairment of the NA-mediated regulation of the spinal nociceptive network would induce exaggerated nociception in PDN. The mechanism may involve a reduced amount of extracellular NA in the spinal cord due to exaggerated NA uptake by overexpressed NETs. The selective enhancement of reduced noradrenergic signals in the spinal cord by inhibiting NA re-uptake might underlie the analgesic effect of DLX in a manner that is dependent on descending NET-expressing noradrenergic fibers which remain intact in PDN.

## Methods

### Preparation of the STZ-induced diabetic model

The manipulation of the animals conformed to the Guiding Principles for the Care and Use of Animals in the Field of Physiological Sciences of the Physiological Society of Japan (1988). The study was approved by the Animal Care Committee of The Jikei University School of Medicine, Tokyo, Japan. Male Wistar rats, weighing 200–230 g, were rendered diabetic by an injection of STZ (50 mg/kg, i.v.) (Sigma-Aldrich, Tokyo, Japan) dissolved in 0.9% sterile saline under deep anesthesia with isoflurane. Age-matched control rats received equal volumes of the vehicle (non-diabetic rats). The animals fasted from the evening prior to the day of STZ administration; they were allowed to feed again after administration of the agent. Diabetes was confirmed one week after injection of STZ by measuring glucose blood levels in samples taken from the tail vein using a OneTouch Ultra blood glucose meter (Johnson & Johnson, Tokyo, Japan). Because 600 mg/dl was the detection limit of the blood glucose meter, the blood glucose levels over 600 mg/dl were defined as 600 mg/dl.

### von Frey filament test

To assess mechanical allodynia, we determined the withdrawal threshold of hind paws to mechanical stimulation using a series of von Frey filaments (North Coast Medical, Inc., Gilroy, CA, USA). We used eight different von Frey filaments ranging from 0.4 g to 15 g. The rats were placed on a metal mesh floor and von Frey filaments were applied from underneath the floor. We estimated the paw withdrawal thresholds by the up-and-down method [70]; we used the mean of right and left paw responses for each rat.

### Hargreaves test

We determined the latency of the hindpaw withdrawal evoked by thermal stimulation using a modified Hargreaves Box (University of California, San Diego, CA, USA) [71]. The rats were placed on a glass floor maintained at 30°C in a clear plastic chamber. We focused a mobile radiant heat source, which was located under the glass floor, onto the plantar surface of the right and left hindpaw. We measured paw withdrawal latencies twice for each hindpaw, and we used the mean of the four values for analysis. Five min of rest was allowed between trials. To prevent tissue damage, we set an automatic cutoff at 20 s.

### Lesions of the noradrenergic system

To induce lesions in the noradrenergic system, we used DSP-4 (Sigma-Aldrich, Tokyo, Japan), which is a selective neurotoxin that preferentially degenerates noradrenergic axons originated from the locus coeruleus [72]. Four weeks after STZ or vehicle injection, the rats received an injection of DSP-4 (50 mg/kg, i.p.) dissolved in 0.9% sterile saline [32] under deep anesthesia with isoflurane. The control rats received equal volumes of saline rather than DSP-4. In a subgroup of STZ rats that received DSP-4, fluoxetine (10 mg/kg, i.p.) (Sigma-Aldrich, Tokyo, Japan), which is a selective 5-HT uptake inhibitor, was administered 30 min before the DSP-4 injection to assess the likely involvement of the effect of DSP-4 on serotoninergic terminals [28]. The control rats received equal volumes of saline rather than fluoxetine.

### Administration of DLX

Six weeks after STZ or vehicle injection, the rats received i.p. injection of DLX (10 mg/kg with of 1% DW solution) (Santa Cruz Biotechnology, Inc., Santa Cruz, CA, USA) under deep anesthesia with isoflurane. The control rats received equal volumes of DW.

### Experimental protocols

The von Frey filament and the Hargreaves tests were performed 1 day before the injection of STZ (n = 30 rats) or vehicle (n = 20) immediately before the 1-day fasting. These tests were performed every week until the 6th week after STZ or vehicle injection. On the 28th day after STZ-treatment (4th week), these tests were performed 60 min before the injection of 1) DSP-4 (n = 10 rats) or 2) saline (n = 10) in STZ-treated or vehicle-treated rats and 3) DSP-4 + fluoxetine (n = 5) or 4) DSP-4 + saline (n = 5) in STZ-treated rats. On the 42nd day after STZ-treatment (6th week), at which time DLX (n = 5 rats) or DW (n = 5) was injected, thermal hyperalgesia was assessed twice at 60 min before and 60 min after the injection. Mechanical allodynia was assessed at 30 min before and 90 min after the injection. The values obtained before and after the injection were termed “pre-DLX” and “post-DLX,” respectively, and the value of “post-DLX” divided by “pre-DLX” was considered to be a measurement for the effect of DLX in these tests. The behavior assessment was made by an experimenter who was blinded to the medication application.

### Measurements of NA and 5-HT

Two hours after the injection of DLX or DW, the rats were sacrificed under deep anesthesia with isoflurane. The lumbar spinal cord was dissected, weighed, immediately frozen in liquid nitrogen and subsequently kept at −80°C. Each lumbar spinal cord was homogenized in 200 μl of 0.2 M perchloric acid containing 100 μM Na_2_-EDTA as an internal standard, and centrifuged at 20000 × *g* at 4°C for 15 min. The supernatants were filtered through a 0.2-μm syringe filter and subsequently kept at pH 3.0 by adding 1 M sodium acetate. The concentration of NA and 5-HT was measured using reverse-phase HPLC with electrochemical detection (SRL, Inc., Tokyo, Japan).

### Confocal microscopic fluorescence immunohistochemistry

For immunohistochemistry, eight STZ (four DSP-4-treated and four saline-treated) and eight vehicle (four DSP-4-treated and four saline-treated) rats at the 6th week after STZ or vehicle injection were anesthetized with sodium pentobarbital (50 mg/kg) and were intracardially perfused with cold phosphate-buffered saline (PBS) subsequently with 4% paraformaldehyde (PFA) in 0.1 M phosphate buffer (pH 7.4). The lumbar (L4–L5) spinal cord segments were removed and post-fixed in 4% PFA overnight at 4°C. After cryoprotection through a graded series of sucrose replacements (10%, 20%, and 30% in PBS) at 4°C, each segment was embedded in OCT Compound (4583, Sakura Finetek, Tokyo, Japan) and stored at −80°C.

The spinal cord segments were cut transversely on a cryostat (CM1850, Leica Biosystems, Tokyo, Japan) into 30-μm-thick sections. The sections were mounted on siliconized slides for immunostaining. Nonspecific labeling was blocked by incubation in 1% normal goat serum and 0.4% Triton X-100 in PBS. After blocking, the sections were incubated overnight at 4°C in the mouse monoclonal antibody to DBH (1:600, EMD Millipore, Billerica, MA, USA) or the NET (1:500, Mab Technologies, Stone Mountain, GA, USA) diluted in blocking solution.

After incubation in the primary antibody cocktail, the sections were rinsed in PBS and incubated in a cocktail of goat anti-mouse Alexa Fluor 488 labeled secondary antibodies (1:400, Invitrogen, Grand Island, NY, USA) for 1 hour at room temperature. The sections were subsequently rinsed in PBS and mounted with Aqua-Poly/Mount (Polysciences, Inc., Warrington, PA, USA). The sections were examined using a confocal microscope (Fluoview FV300, Olympus Corporation, Tokyo, Japan). The control sections were processed with the primary antibody omitted from the primary antibody cocktail; in all cases, only the labeling with the secondary fluorescent antibodies corresponding to the non-omitted primary antibody was observed.

### Quantification of DBH- and NET-positive fibers in the lumbar spinal dorsal horn

The areas of the DBH- and NET-immunoreactive pixels in projection confocal images were calculated by fluorescence thresholding with maximum entropy method (NIH ImageJ; Bethesda, MD, USA) and normalized by the area of the dorsal horn gray matter (either side of transverse sections dorsal to the ventromost edge of the white matter at the midline). Each projection confocal image was created using 16 optical sections with a 2.8-μm interval (10× objective; 1024 × 1024 pixels) and 18 sections with a 1.4-μm interval (20× objective; 1024 × 1024 pixels) for DBH and NET, respectively. After the primary measurement by an author, other authors who were blinded to the medication treatments re-examined the confocal images and results of the evaluation in a blinded manner.

### Data and statistical analysis

Values are expressed as the mean values ± standard error of the mean (SEM). The statistical comparisons were made using one-way analysis of variance (ANOVA) for repeated-measures followed by Bonferroni post hoc test for multiple comparisons of the time course, Wilcoxon signed-rank test for between pre-DLX and post-DLX comparisons, Kruskal-Wallis one-way ANOVA for the inter-group multiple comparisons and Mann–Whitney *U-*test for other comparisons between two groups. Differences with a probability (*P*) less than 0.05 were considered to be statistically significant.

## Abbreviations

5-HT: Serotonin; BDNF: Brain-derived neurotrophic factor; DBH: Dopamine-beta-hydroxylase; DLX: Duloxetine; DM: Diabetes mellitus; DSP-4: N-(2-chloroethyl)-N-ethyl-2-bromobenzylamine; DW: Distilled water; HPLC: High-performance liquid chromatography; MHPG: 3-methoxy-4-hyroxyphenylglycol; NA: Noradrenaline; NET: Norepinephrine transporter; PBS: Phosphate-buffered saline; PDN: Painful diabetic neuropathy; PFA: Paraformaldehyde; SNRI: Serotonin and noradrenaline re-uptake inhibitor; SSRI: Selective serotonin re-uptake inhibitor; STZ: Streptozotocin; TH: Tyrosine hydroxylase.

## Competing interests

The Department of Neuroscience, of which FK is the Director, received a research grant from Shionogi Co. LTD.

## Authors’ contributions

JK and YT carried out all experiments and data analyses. JK, YT, AMW and FK wrote the text, KU and FK designed the study. All 5 authors participated in the discussion. All authors read and approved the final manuscript.
